# Targeted cultivation of diatoms in mariculture wastewater by nutrient regulation and UV-C irradiation

**DOI:** 10.3389/fmicb.2024.1371855

**Published:** 2024-03-13

**Authors:** Jiacong Shen, Xiafei Zheng, Minhai Liu, Kui Xu, Lin He, Zhihua Lin

**Affiliations:** ^1^Ninghai Institute of Mariculture Breeding and Seed Industry, Zhejiang Wanli University, Ningbo, China; ^2^Hubei Key Laboratory of Edible Wild Plants Conservation and Utilization, Hubei Engineering Research Center of Special Wild Vegetables Breeding and Comprehensive Utilization Technology, College of Life Sciences, Hubei Normal University, Huangshi, China

**Keywords:** diatom, *Chaetoceros muelleri*, mariculture wastewater, silicate, UV-C irradiation

## Abstract

Mariculture wastewater poses environmental challenges due to pollution and eutrophication. Targeted cultivation of diatoms in wastewater can help alleviate these issues while generating beneficial algae biomass, however reliable operating methods are lacking. We proposed a novel method for treating mariculture wastewater that employed UV-C irradiation and nutrient regulation to achieve targeted diatom cultivation. This study first examined growth of four diatom species (*Nitzschia closterium*, *Chaetoceros muelleri*, *Cyclotella atomus*, and *Conticribra weissflogii*) in mariculture wastewater. *C. muelleri* and *C. weissflogii* demonstrated better adaptability compared to *N. closterium* and *C. atomus*. Additionally, the growth and nutrient utilization of *C. muelleri* were studied under varying concentrations of silicate, phosphate, ammonium, and trace elements in wastewater. Optimal growth was observed at 500 μmol/L silicate, 0.6 mg/L phosphate, and 4 mg/L ammonium. Ammonium proved to be a more effective nitrogen source than urea and nitrate in promoting growth at this low level. Surprisingly, trace element supplementation did not significantly impact growth. Finally, this study utilized UV-C irradiation as a pre-treatment method for wastewater prior to nutrient adjustment, significantly enhancing the growth of *C. muelleri*. Overall, this study provides guidance on regulating key nutrients and pre-treatment method to optimize diatom biomass production from mariculture wastewater. This approach not only addresses environmental challenges associated with mariculture but also contributes to sustainable aquaculture practices through the recovery of valuable aquatic resources.

## 1 Introduction

Mariculture, also known as marine aquaculture, is a rapidly growing food-producing industry globally, effectively addressing the supply–demand gap for aquatic food (Food and Agriculture Organization (FAO) of the United Nations, 2022). Beyond optimizing diets through high-protein seafood provision, mariculture can reshape marine fisheries and improve coastal rural incomes (Xie et al., 2013; Zou and Huang, 2015). However, large-scale mariculture expansion places significant environmental pressures on the natural environment (Joesting et al., 2016; Bambaranda et al., 2019). Aquaculture wastewater containing residual feed, organism waste, drugs, and debris causes substantial coastal pollution and eutrophication, hindering industry and ecosystem development (Yu and Yin, 2019). According to the Chinese Marine Ecological Environment Bulletin in 2022, the areas of water classified as inferior to Class IV quality standards due to excessive levels of inorganic nitrogen and phosphate were 24,580 and 6,070 square kilometers, respectively (Ministry of Ecology and Environment (MEE) of the People's Republic of China, 2023). Moreover, China encountered 67 red tide events spanning 3,328 square kilometers in 2022 (Ministry of Ecology and Environment (MEE) of the People's Republic of China, 2023). Consequently, proper treatment of mariculture wastewater is imperative for sustainable marine aquaculture.

The primary goal of wastewater treatment is to reduce pollutants and achieve nutrient recycling (Ramesh Kumar et al., 2019). Since aquaculture wastewater contains fewer toxic substrates than industrial wastewater, it presents a higher potential capacity for recovery and sustainable reuse (Puyol et al., 2017). However, our investigation found that the typical lease term for aquaculture ponds is only 3–5 years in eastern China, which deters farmers from investing in constructing wastewater treatment facilities. Recently, centralized mariculture wastewater treatment has become a trend in China, especially in large-scale concentrated pond areas and industrial mariculture garden areas (Zheng et al., 2022). Non-feeding shellfish culture is commonly employed for this purpose, although its efficiency in removing inorganic nutrients is limited (Rose et al., 2015). An optimal approach would involve stimulating phytoplankton growth before the wastewater enters into the shellfish culture sector. Our team previously proposed a novel method for treating mariculture wastewater, which combined microalgae, shellfish, macroalgae, and microbial treatment (Supplementary Figure S1). We suggest adding a diatom targeted culture unit prior to shellfish filtration to improve inorganic nutrient removal and recovery. However, a key technical challenge is how to regulate the algal community structure to maximize benefits for shellfish consumption.

Diatoms, accounting for 40% of oceanic primary production, are essential for bivalve culture due to their nutritional value (González-González et al., 2019). They are highly valuable for their rapid growth, easy maintenance, and suitability as an aquacultural feed (Minggat et al., 2021). Additionally, their bioremediation capability through efficient wastewater pollutant removal has been recognized (Zhi et al., 2019; Tanaka et al., 2020; Minggat et al., 2021). Diatom growth is influenced by various environmental factors such as temperature, light, salinity, pH, CO_2_ concentration, and particularly nutrient availability (Iwasaki et al., 2021). However, centralized mariculture wastewater presents a challenge due to its complexity, originating from various ponds and factories. Optimizing nutrient structures in such wastewater to promote diatom growth is crucial. Besides, the mariculture wastewater also contains a diverse mix of algae and bacteria, leading to both synergistic and antagonistic interactions (Zheng et al., 2023). A major challenge in the targeted cultivation of diatom is the competition for nutrients with native microorganisms (Diner et al., 2016; Zhou et al., 2017). While lab-scale studies often use autoclave sterilization (Joesting et al., 2016; Xing et al., 2018; Bambaranda et al., 2019; Saxena et al., 2022), large-scale applications of targeted diatom culture require practical methods to minimize this competition. UV-C irradiation has been identified as an effective technique to eliminate algae and bacteria in wastewater, showing promise for large-scale use (Tao et al., 2010; Passero et al., 2014).

The objective of this study was to develop a technical method for the targeted cultivation of diatoms in mariculture wastewater by regulating nutrients and microorganisms. Initially, we assessed the growth of four diatom species (*Nitzschia closterium*, *Chaetoceros muelleri*, *Cyclotella atomus*, and *Conticribra weissflogii*) in mariculture wastewater, finding *C. muelleri* and *C. weissflogii* to be the most adaptable. We then manipulated concentrations of silicate, nitrogen, phosphate, and trace elements to optimize diatom growth and nutrient utilization. The study also explored the combined effects of UV-C irradiation and nutrient regulation on diatom growth. Based on our experimental findings, an *in-situ* diatom targeted culture technique utilizing mariculture wastewater was developed. This technology can be applied to mariculture wastewater treatment and achieve resource recovery in combination with shellfish culture.

## 2 Materials and methods

### 2.1 Measurement of chlorophyll content and water quality parameters

The mariculture wastewater used in this study was collected from the drainage channel of the She Pan Tu mariculture garden (29.1538N, 121.5056E) in Ninghai, Zhejiang, China. This 2,141-acre garden encompassed diverse aquaculture practices, including pond aquaculture, aquaculture integrated with photovoltaic power generation, and industrialized aquaculture, thus contributing to the varied composition of the wastewater. Pond aquaculture and photovoltaic-integrated aquaculture primarily cultured Pacific white shrimp (*Penaeus vannamei*), razor clam (*Sinonovacula constricta*), blood clam (*Tegillarca granosa*), and green crab (*Scylla serrata*), whereas industrialized aquaculture focused on Pacific white shrimp and black tiger prawn (*Penaeus monodon*). In each experiment, the concentrations of silicate, phosphate, ammonium, nitrite, and nitrate in the mariculture wastewater were measured with various spectrophotometric assays according to National Standards of the PRC (GB/T 12763.4–2007, 2007). The measurement methods included the silico-molybdenum blue method for silicate, phosphor molybdenum blue spectrophotometry for phosphate, hypobromite oxidation for ammonium, diazotization azo method for nitrite, and cadmium-copper reduction method for nitrate. Additionally, the concentration of chlorophyll *a* was measured using a PHYTO-PAM-II phytoplankton classification fluorometer (Walz GmbH, Effeltrich, Germany).

### 2.2 Algae choice

For this study, four diatom species were selected based on their common use as bait algae in shellfish nurseries. The diatoms *Nitzschia closterium*, *Cyclotella atomus*, and *Conticribra weissflogii* were acquired from Professor Pengfei Cheng at Ningbo University, and *Chaetoceros muelleri* was obtained from Ninghai Bei Bei Le Aquaculture Company. These species were chosen to assess their growth potential in mariculture wastewater compared to the f/2 medium, a standard culture medium for marine microalgae (Guillard and Ryther, 1962). Initially, mariculture wastewater was collected from the drainage channel, immediately transported to the lab, and sterilized by autoclaving at 121°C for 30 min. The nutrient status of the wastewater was then analyzed (Supplementary Table S1). To ensure consistency, the four diatom species in the logarithmic growth phase were diluted to a same chlorophyll *a* concentration. Then, 15 mL aliquots of each species were inoculated into both the wastewater and f/2 medium, with each treatment replicated three times. Cultures were incubated in a smart light incubator (GXZ-380A, Ningbo Jiangnan Instrument Factory) at 25 ± 1°C with 4000 lux irradiance under a 12 h:12 h light/dark cycle. Diatom growth was monitored over 7 days by daily chlorophyll *a* concentration measurements.

### 2.3 Regulation of silicate

This part of the study focused on determining the optimal silicate concentration for the growth of *C. muelleri*. Five silicate levels (50, 100, 500, 1,000, 4,000 μmol/L) of Na_2_SiO_3_·9H_2_O were evaluated, along with blank and f/2 medium controls, each in triplicate. Mariculture wastewater, collected from the drainage channel, was immediately transported to the lab for autoclaving sterilization at 121°C for 30 min. After assessing its nutritional content (Supplementary Table S1), silicate concentrations were adjusted in the experimental groups. Subsequently, 15 mL of *C. muelleri* from the logarithmic growth phase was inoculated into the wastewater, mixed thoroughly, and incubated at 25 ± 1°C in a light incubator with 4,000 lux irradiance, following a 12 h:12 h light/dark cycle. Chlorophyll *a* concentration and silicate content in the culture medium were measured daily and bi-daily, respectively. The experiment was monitored for 7 days.

### 2.4 Regulation of phosphate

Mariculture wastewater, collected from the drainage channel, was immediately transported to the lab and sterilized. Its nutritional content was analyzed (Supplementary Table S1). In the first experiment, five concentration gradients (0.2, 0.4, 0.6, 0.8, and 1.0 mg/L) of phosphate were used, as well as blank and f/2 medium controls, with three replicates for each group. In all experimental groups and blank control, the silicate concentration was regulated to 500 μmol/L. In the second experiment, a total of five concentration gradients (0.6, 0.9, 1.2, 1.5, and 1.8 mg/L) were used, along with blank and f/2 medium controls, with three replicates for each group. In all experimental groups and blank control, the ammonium and silicate were regulated to 4 mg/L and 500 μmol/L, respectively. The phosphate concentration in wastewater was adjusted according to the experimental design. Subsequently, 15 mL of *C. muelleri* from the logarithmic growth phase was inoculated into the wastewater, mixed thoroughly, and incubated at 25 ± 1°C in a light incubator with 4,000 lux irradiance, following a 12 h:12 h light/dark cycle. The concentration of chlorophyll *a* was measured daily, while the phosphate content of the culture medium was determined every 2 days for 7 days.

### 2.5 Regulation of nitrogen

Mariculture wastewater, collected from the drainage channel, underwent sterilization for subsequent use. Its nutritional content was analyzed (Supplementary Table S1). NH_4_Cl was used as the nitrogen source, with five concentration gradients established at 2, 4, 6, 8, and 10 mg/L, as well as blank control and f/2 medium controls, with three replicates for each group. In the experimental group and blank control, the silicate and phosphate were regulated to 500 μmol/L and 0.6 mg/L, respectively. Subsequently, 15 mL of *C. muelleri* from the logarithmic growth phase was inoculated into the wastewater, mixed thoroughly, and incubated at 25 ± 1°C in a light incubator with 4,000 lux irradiance, following a 12 h:12 h light/dark cycle. The concentration of chlorophyll *a* was measured daily, while the ammonium and nitrite concentrations were determined every 2 days over a period of 7 days.

### 2.6 Comparison of different nitrogen sources

In this experiment, the effects of three nitrogen sources, namely urea, ammonium, and nitrate, on diatom growth were examined. First, the collected mariculture wastewater was sterilized and its nutritional content was determined for subsequent use (Supplementary Table S1). Each nitrogen source was added at a final concentration of 4 mg/L. The experiment included a blank control and an f/2 medium control. All the groups had three replicates for each group. In both the experimental and blank control groups, silicate and phosphate levels were regulated to 500 μmol/L and 0.6 mg/L, respectively. Subsequently, 15 mL of *C. muelleri* from the logarithmic growth phase was inoculated into the wastewater, mixed thoroughly, and incubated at 25 ± 1°C in a light incubator with 4,000 lux irradiance, following a 12 h:12 h light/dark cycle. The concentration of chlorophyll *a* was measured daily, while the ammonium, nitrite, and nitrate contents in the medium were measured every 2 days. The experiment was monitored for a duration of 7 days.

### 2.7 Regulation of trace elements

The collected mariculture wastewater was sterilized and its nutritional content was determined for subsequent use (Supplementary Table S1). The experimental gradients of trace elements were established at concentrations of 0.5, 1, 2, 4, and 8 mL/L in the wastewater, along with a blank control and an f/2 medium control, with three replicates per group. The 1 L trace element solution comprised 3.15 g FeCl_3_·6H_2_O, 4.36 g Na_2_EDTA·2H_2_O, 9.8 mg CuSO_4_·5H_2_O, 6.3 mg NaMoO_4_·2H_2_O, 22.0 mg ZnSO_4_·7H_2_O, 10.0 mg CoCl_2_·6H_2_O, and 180.0 mg MnCl_2_·4H_2_O. In both the experimental and blank control groups, the silicate, phosphate, and nitrogen were regulated to 500 μmol/L, 0.6 mg/L, and 4 mg/L, respectively. Trace elements were added according to the experimental design. Subsequently, 15 mL of *C. muelleri* from the logarithmic growth phase was inoculated into the wastewater, mixed thoroughly, and incubated at 25 ± 1°C in a light incubator with 4,000 lux irradiance, following a 12 h:12 h light/dark cycle. Chlorophyll *a* concentration was measured daily for 7 days.

### 2.8 Combination of UV irradiation and nutrient regulation

The mariculture wastewater, collected from the drainage channel, was initially subjected to different pre-treatments, including autoclave sterilization (121°C, 30 min), UV-C irradiation (254 nm, 20 W, 10 min), and a control group without any treatment. Each group consisted of three replicates. Then the nutritional content of wastewater was analyzed (Supplementary Table S1). Following the pre-treatment, all groups were subjected to the same nutrient regulation strategy with the silicate, phosphate, and ammonium adjusted to 500 μmol/L, 0.6 mg/L, and 4 mg/L, respectively. The mariculture wastewater was placed in the dark for 24 h after pretreatments. Subsequently, 15 mL of *C. muelleri* from the logarithmic growth phase was inoculated into the wastewater, mixed thoroughly, and incubated at 25 ± 1°C in a light incubator with 4,000 lux irradiance, following a 12 h:12 h light/dark cycle. The chlorophyll *a* content of diatom and green algae was then examined using the PHYTO-PAM-II phytoplankton classification fluorometer for 7 days.

### 2.9 Microbial community analysis

During the UV irradiation experiment, phytoplankton samples were collected from both raw and 7-day cultivated wastewater using 0.22 μm polyethersulfone (PES) filters (Pall, United States). The phytoplankton DNA on the filters was extracted using the PowerWater DNA Isolation Kit (MoBio Laboratories, Carlsbad, United States). For the microalgae community, the V4 region of the 18S rRNA gene was amplified using the TAReuk454FWD1 and TAReukREV3 primers (Stoeck et al., 2010), following the PCR procedure in our previous study (Zheng et al., 2021). For the bacterial community, the V3-V4 region of the 16S rRNA gene was amplified by the 338F/806R primer pair, with a PCR program consisting of initial denaturation at 95°C for 5 min, followed by 25 cycles of denaturation, annealing, and extension at 95°C for 30 s, 50°C for 30 s, and 72°C for 40 s, respectively, and a final extension at 72°C for 7 min. The PCR products were sequenced on an Illumina NovaSeq 6000 PE 250 platform (Biomarker, China). Quality control and removal of chimeric sequences were conducted using USEARCH v.11 software (Edgar, 2010). A zero-radius operational taxonomic unit (ZOTU) table was generated using the USEARCH denoising algorithm. Representative sequence and taxonomy classification were identified for each ZOTU and aligned against Protist Ribosomal Reference (PR2) database 4.14 (18S rRNA gene) (Guillou et al., 2013) and RDP v18 database (16S rRNA gene) (Cole et al., 2014). The raw sequence data are available in the NCBI database under BioProject accession numbers PRJNA1058243 (18S) and PRJNA1058104 (16S).

### 2.10 Statistical analysis

The experimental data were analyzed using one-way ANOVA, followed by Duncan’s test for pairwise comparisons using the GraphPad Prism 9. All statistical analyses were conducted with a significance level set at *p* < 0.05.

## 3 Results and discussion

### 3.1 Growth comparison of four diatom species in mariculture wastewater

**Figure 1:**
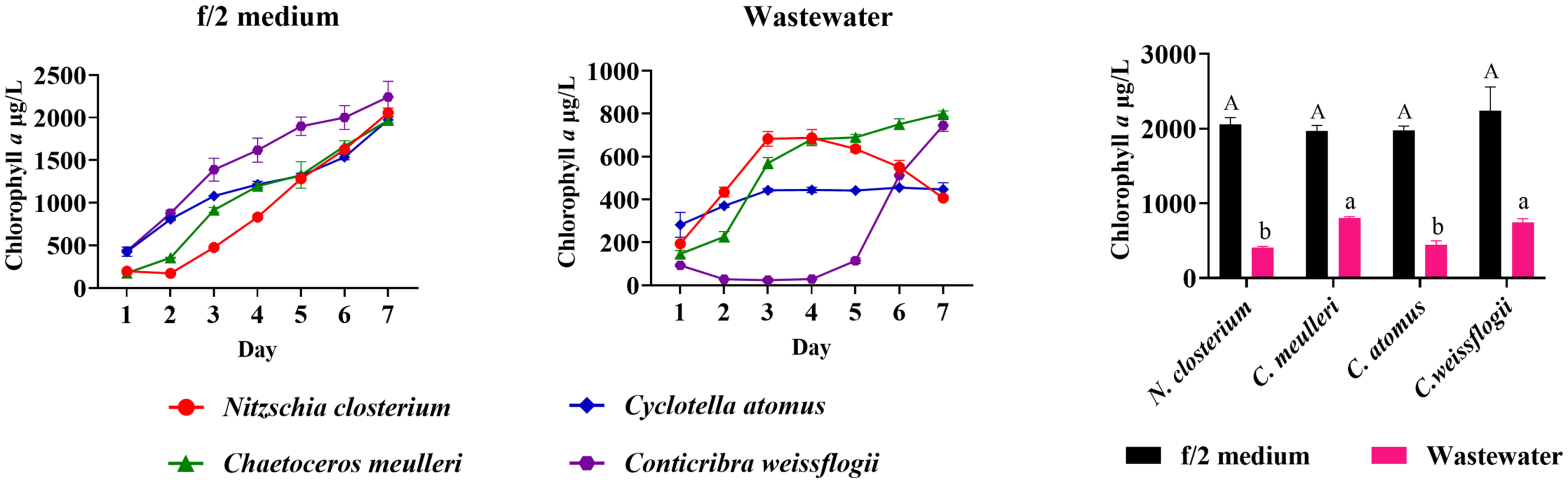
Comparison of growth and final biomass of *Nitzschia closterium*, *Chaetoceros meulleri*, *Cyclotella atomus*, and *Conticribra weissflogii* in f/2 medium and wastewater. Different letters indicate statistical differences at 0.05 significance level (One-Way ANOVA).

In order to assess the adaptability of four diatom species in the mariculture wastewater, the growth characteristics of four diatom species in the wastewater and f/2 medium were compared. The results indicated no significant growth difference among the species in f/2 medium (Figure 1). However, their growth in mariculture wastewater significantly differed from that in f/2 medium (*p* < 0.05, Figure 1). In the first 3 days, *N. closterium* showed a marked increase in growth, followed by a decrease. *C. atomus* exhibited a similar trend, but its growth was slow, with no significant biomass increased after 3 days. *C. muelleri* and *C. weissflogii* displayed better adaptability in the mariculture wastewater, with *C. muelleri* exhibiting a consistent increase in biomass. *C. weissflogii* initially declined but recovered after 4 days, eventually equalling the final biomass of *C. muelleri*. Given its robust growth in wastewater, as also supported by previous studies (Karthikeyan et al., 2013; Hemalatha et al., 2014; Saxena et al., 2022), *C. muelleri* was chosen for subsequent targeted diatom culture experiments. Based on our findings, although the nutrients in the wastewater supported a certain growth capacity for the diatoms, it was insufficient to ensure optimal growth compared to the f/2 medium. Therefore, adjusting the nutrient composition of mariculture wastewater is crucial for optimizing diatom growth.

### 3.2 Diatom growth after silicate regulation in mariculture wastewater

**Figure 2:**
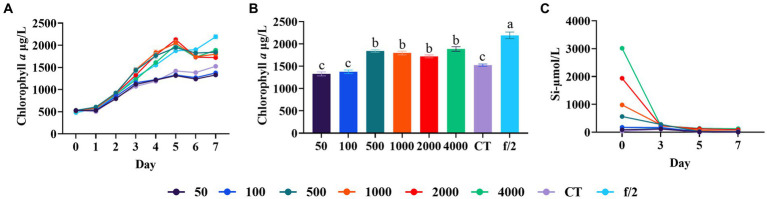
Growth of *C. meulleri* under different silicate concentrations. **(A)** Growth trend of *C. meulleri* under different silicate gradients. **(B)** Silicate concentration changed during the experiment. **(C)** Comparison of diatom biomass on day 7. Different letters indicate statistical differences at 0.05 significance level (One-Way ANOVA).

Silicon is abundant in the earth’s crust and plays a crucial role in diatoms, as they utilize silicon to build their cell walls. Marine diatoms primarily absorb silicon in the form of Si(OH)_4_ for cell wall biosynthesis, however the solubility of Si(OH)_4_ is relatively low in seawater (<2 mM) (Martin-Jézéquel et al., 2000). Arsad et al. (2019) examined the culture of *Haslea ostrearia* through different mediums, and found the seawater added with silicate showed better growth. Saxena et al. (2022) found that the addition of inductively coupled plasma nanosilica (ICP-SiO_2_) improved diatoms to utilize nutrients from aquaculture wastewater. Hemalatha et al. (2012, 2014) reported that a high chlorophyll *a* content of *Chaetoceros simplex* was obtained when the SiO_3_^2−^ was 265 μmol/L. In our experiment, we observed a significant improvement in diatom growth with the addition of 500 μmol/L silicate compared to control and lower silicate levels (50 and 100 μmol/L) (Duncan’s test, *p* < 0.05, Figure 2A). However, further increasing the silicate concentration to 1,000–4,000 μmol/L did not yield additional biomass enhancement (Duncan’s test, *p* > 0.05, Figure 2B). In all experimental groups, the silicate concentration decreased to below 30 μmol/L after a 7-day culture cycle. This finding is consistent with a previous study, which has shown that 1.5 mM Si (Na_2_SiO_3_) can be consumed within 5 days by *Cyclotella* sp. (Ozkan and Rorrer, 2017). These results indicate a high silicate utilization rate and demand in *C. muelleri*.

Although silicate addition significantly improved biomass in our experiment, the total biomass was still lower than that observed in the f/2 medium control (*p* < 0.05, Figure 2C). This suggests that there may be other limiting nutrient factors affecting the growth of *C. muelleri*.

### 3.3 Diatom growth after phosphate regulation in mariculture wastewater

**Figure 3:**
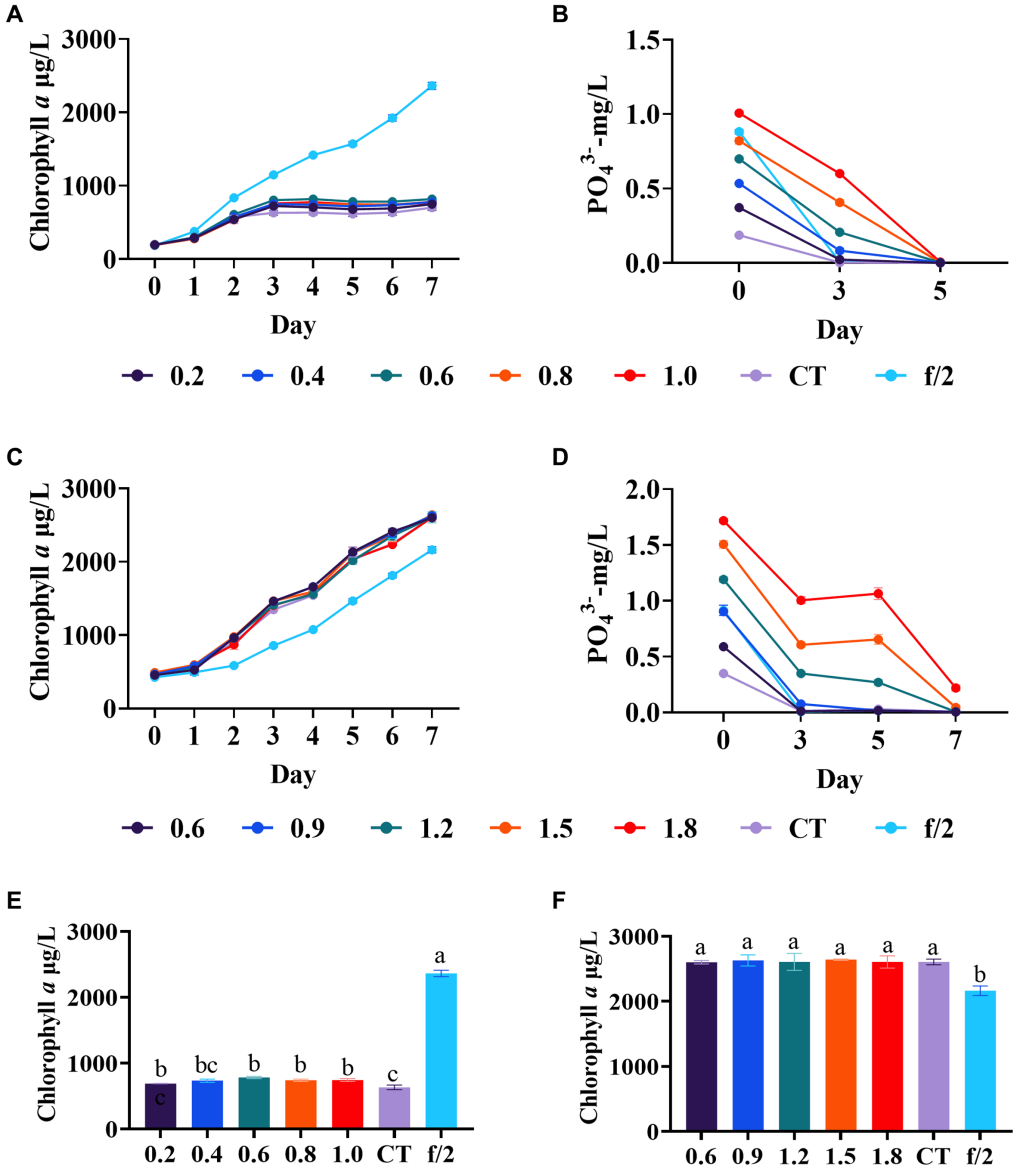
Diatom growth after phosphate regulation in wastewater. **(A)** Growth trend of *C. meulleri* under low level phosphate gradients; **(C)** Growth trend of *C. meulleri* under high level phosphate gradients. **(B,D)** Phosphate concentration changed during the experiment. **(E,F)** Comparison of diatom biomass on day 7. Different letters indicate statistical differences at 0.05 significance level (One-Way ANOVA).

In the first phosphate regulation experiment, diatom growth in all experimental groups plateaued after 3 days, showing no significant difference in the final biomass among these groups (Figure 3A). However, a significant difference was found between the blank control and experimental groups (0.6–1.0 mg/L phosphate) (*p* < 0.05, Figure 3E), with the final biomass in experimental groups was significantly lower than in the f/2 medium control. The phosphate concentration in all experimental groups was nearly reduced to 0.01 mg/L after 5 days (Figure 3B). It is noteworthy that the ammonium concentration in the mariculture wastewater used in this experiment was relatively low with only 0.3 mg/L (Supplementary Table S1), which may also be a contributing factor to the poor overall growth of *C. muelleri*.

Therefore, to exclude the potential influence of low nitrogen content on the poor growth of *C. muelleri*, a second phosphate regulation experiment was conducted. The gradients were adjusted based on the high phosphate utilization rate in the first experiment. Based on the results of silicate regulation experiments, the ammonium and silicate were adjusted to 4 mg/L and 500 μmol/L, respectively. In this experiment, the initial concentration of phosphate is 0.36 mg/L in the control. In contrast to the first experiment, the diatoms in all the experimental groups exhibited robust growth, with higher biomass compared to the f/2 medium control (Figure 3C). However, no significant growth differences were observed between the groups (Figure 3F). The results also revealed that the utilization rate of phosphate by *C. muelleri* is still very high (Figure 3D). On day 5, the phosphate content of the experimental group with a concentration below 0.9 mg/L was reduced to below 0.01 mg/L, which was similar to the results of the first experiment. Hemalatha et al. (2014) found *Chaetoceros simplex* obtained maximum growth with the phosphate at 72.4 μM (2.2 mg/L) for 16 days. In the previous study, the dry biomass of *C. muelleri* with the phosphate at 0.6 and 4.46 mg/L had no significant difference (Lovio-Fragoso et al., 2019). Our results indicated *C. muelleri* requires limited phosphate, likely around 0.6 mg/L, which can support growth while minimizing environmental phosphorus discharge.

### 3.4 Diatom growth after ammonium nitrogen regulation in mariculture wastewater

**Figure 4:**
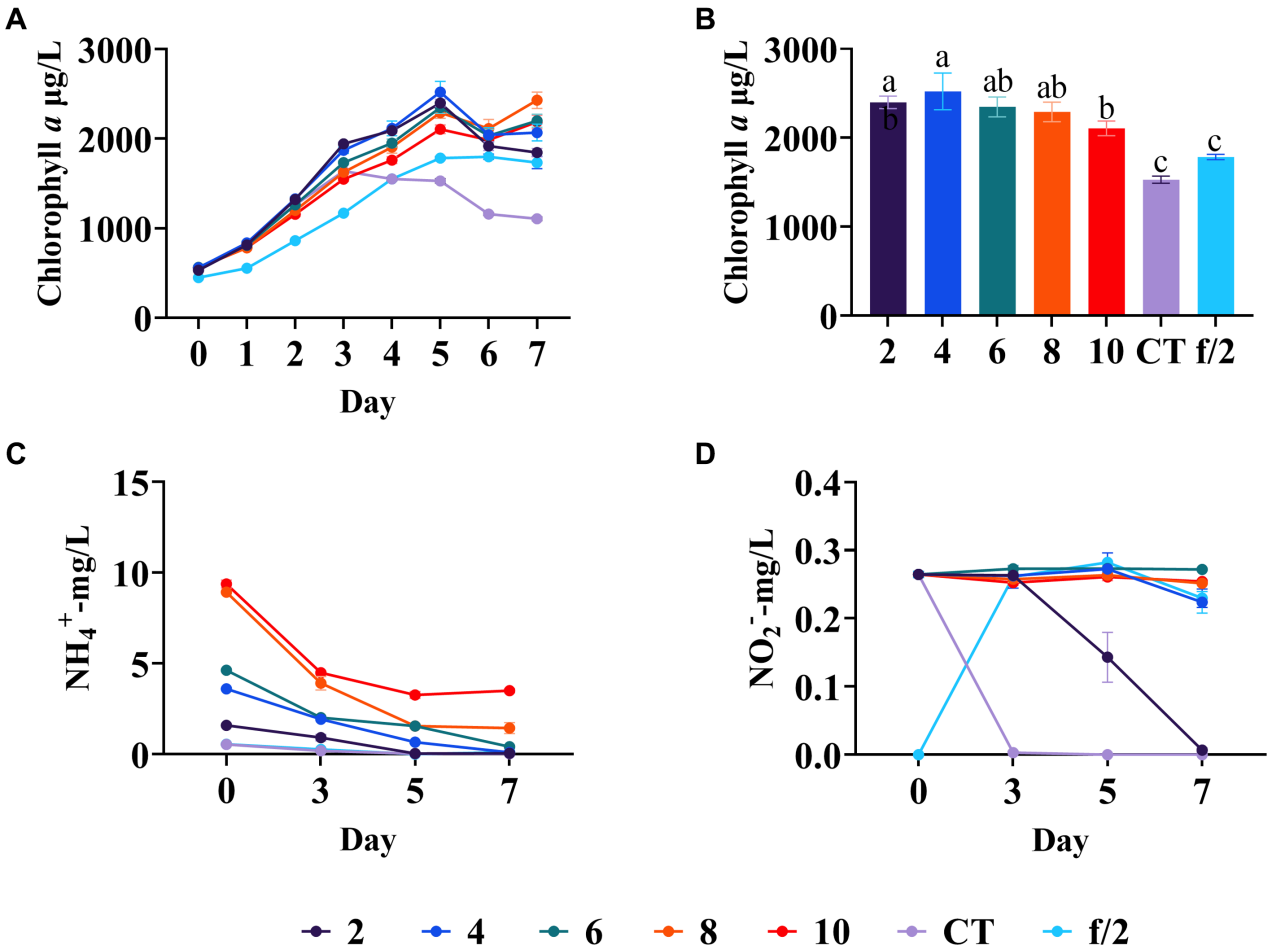
Diatom growth under different concentrations of ammonium nitrogen in wastewater. **(A)** Growth trend of *C. meulleri* under different ammonium nitrogen gradients. **(B)** Comparison of diatom biomass on day 5. **(C)** Ammonium concentration changed during the experiment. **(D)** Nitrite concentration changed during the experiment. Different letters indicate statistical differences at 0.05 significance level (One-Way ANOVA).

Ammonium is a key nitrogen pollutant in mariculture wastewater (Hargreaves, 1998). Reis Batista et al. (2015) indicated that ammonium nitrogen in the media leads to a longer stationary phase, higher dry weight production, and higher lipid content of *C. muelleri* compared to nitrate nitrogen. In this study, the silicate and phosphate regulation experiments all indicated that ammonium seems to be critical for the growth of diatoms. Our study examined ammonium nitrogen on diatom growth, finding the diatom biomass in all the experimental groups was higher than in the blank control and the f/2 medium control. The diatom biomass in all experimental groups increased from day 0 to day 5, and exhibited a downward trend from day 6 (Figure 4A). Notably, the diatom biomass increased from the 2 mg/L to 4 mg/L ammonium nitrogen group but decreased from the 4 mg/L to 10 mg/L ammonium nitrogen group on day 5 (Figure 4B). Moreover, ammonium concentrations in 2 mg/L and 4 mg/L groups, as well as the blank and f/2 medium control groups, all dropped below 0.1 mg/L on day 5 (Figure 4C). Additionally, diatoms began to use nitrite nitrogen in the control and 2 mg/L ammonium groups from day 3 to day 7, indicating the shortage of ammonium nitrogen in these groups (Figure 4D). Our results showed ammonium with 10 mg/L had adverse effects on the growth of *C. muelleri* (*p* < 0.05, Figure 4B). A previous study also showed that ammonium with 1.18 mM (16.5 mg/L) had toxicity to some algae, but the toxicity disappeared when the concentration decreased to 0.3–0.4 μM (4.2–5.6 mg/L) (Lourenço et al., 2002). Considering the diatom growth patterns and ammonium utilization efficiency, the optimal ammonium concentration for mariculture wastewater appears to be 4 mg/L.

### 3.5 Comparison of different nitrogen sources on the diatom growth in mariculture wastewater

**Figure 5:**
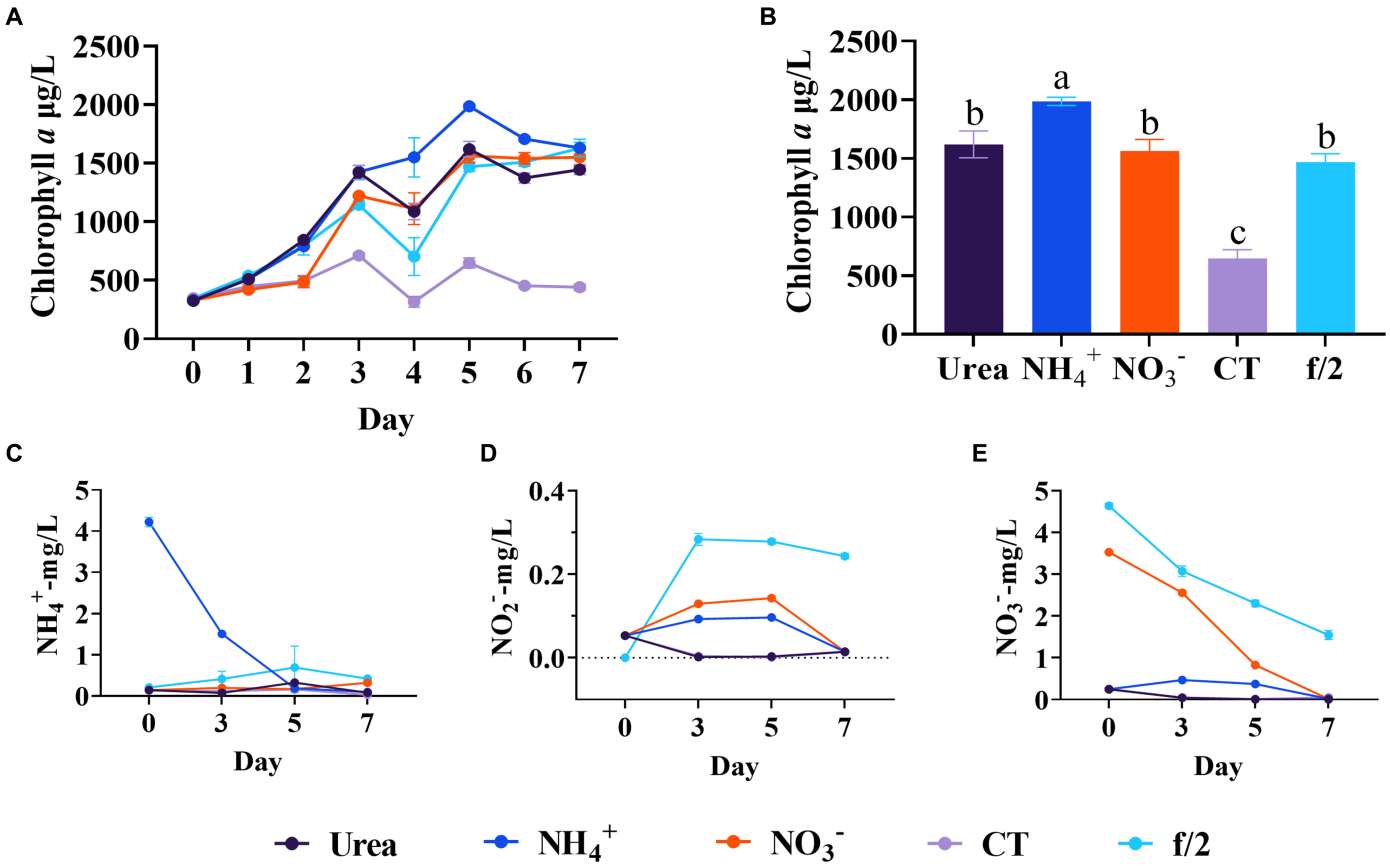
Comparison of different nitrogen sources on the diatom growth. **(A)** Growth trend of *C. meulleri* with different nitrogen sources. **(B)** Comparison of diatom biomass on day 5. **(C)** Ammonium concentration changed during the experiment. **(D)** Nitrite concentration changed during the experiment. **(E)** Nitrate concentration changed during the experiment. Different letters indicate statistical differences at 0.05 significance level (One-Way ANOVA).

Based on the results of the ammonium regulation experiments, this experiment further compared the effects of different nitrogen sources on the growth of *C. muelleri*. Diatom abundance generally increased from day 0 to day 5, but showed a decreasing trend since day 6 (Figure 5A). On day 5, the diatom biomass was the highest in the ammonium group, followed by the urea and nitrate groups, and then the f/2 medium group. There was no significant biomass difference between the urea and nitrate groups on day 5 (*p* < 0.05, Figure 5B). Our study found that *C. muelleri* with ammonium source had the highest peak in the exponential phase, but the final biomass had no significant difference among the groups with different nitrogen sources in the stationary phases. Liang et al. (2006) found that nitrogen source had no significant effect on the final cell density of *C. muelleri* at a concentration of 11 mg/L. Reis Batista et al. (2015) also found nitrate source will more easily lead to collapse, and ammonium source has more longer stationary phase and leads to higher biomass finally with a concentration of 16.5 mg/L. Chen et al. (2023) found urea was more favorable than ammonium for the growth of *Chaetoceros* sp. when nitrogen concentration was 750 mg/L. It is important to note that high concentrations of ammonium have adverse effects on diatom growth. In our study, the ammonium concentration exceeding 10 mg/L significantly inhibited diatom growth. In the previous studies, the ammonium concentration is all above 10 mg/L, which can be a critical factor influencing the results.

During the experiment, the diatoms efficiently utilized both ammonium and nitrate nitrogen, lowering their concentrations in the wastewater (Figures 5C–E). This high efficiency in nitrogen uptake helps explain why the final biomass of diatoms showed no significant differences among the different nitrogen sources. The nitrite concentration initially increased and then decreased in the ammonium and nitrate groups, ultimately being fully utilized by the end of the experiment. The nitrate concentration in the ammonium and urea groups remained at a low level throughout the experiment. We acknowledge that at higher nitrogen concentrations, e.g., 10 mg/L or above, nitrate and urea nitrogen can indeed contribute to higher diatom biomass (Karthikeyan et al., 2013). However, it is not ideal to regulate the nitrate or urea to high concentrations in mariculture wastewater treatment. Manipulating high nitrogen concentrations in the algae pool requires more hydraulic retention time, which is impractical. Overall, our study revealed that ammonium is a beneficial nitrogen source when adjusted to a concentration of 4 mg/L.

### 3.6 Diatom growth after trace element regulation in mariculture wastewater

**Figure 6:**
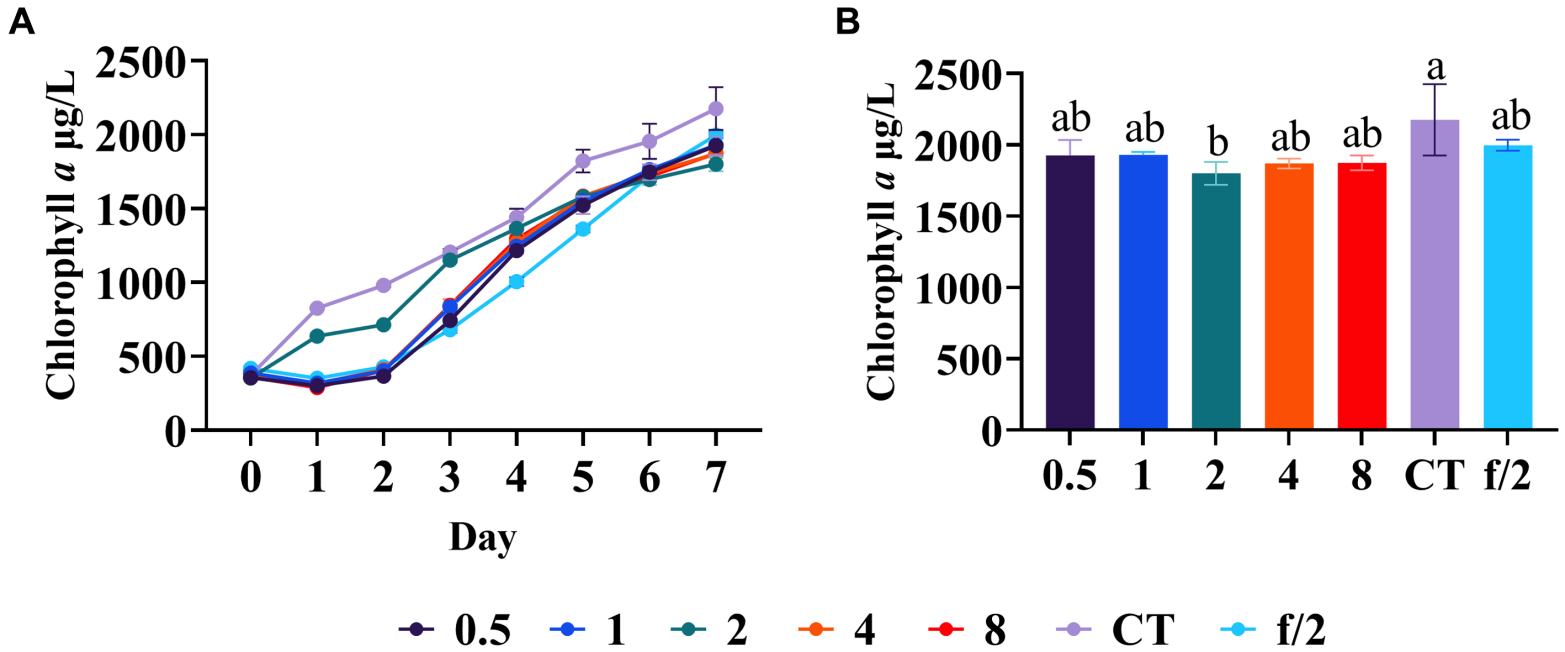
Diatom growth after trace element regulation. **(A)** Growth trend of *C. meulleri* under different gradients of trace element. **(B)** Comparison of diatom biomass on day 7. Different letters indicate statistical differences at 0.05 significance level (One-Way ANOVA).

In order to investigate the necessity of artificially supplementing trace elements to promote the growth of *C. muelleri*, an experiment was conducted in which varying concentrations of trace elements were added into mariculture wastewater and the resulting diatom growth was compared. Specifically, the growth trend of *C. muelleri* and the diatom biomass on day 7 were examined. Our results indicated that the supplementation of trace elements did not improve the diatom growth (Figure 6). Furthermore, there was no significant difference among the experimental groups (Figure 6), suggesting that there was no shortage of trace elements in aquaculture wastewater. It is worth considering that the formulated feed used in aquaculture contains various trace elements (Sarkar et al., 2022). Besides, aquaculture sediment can serve as a source of trace elements and may release trace elements into the overlaying water (Chen et al., 2007). Additionally, the frequent application of fertilizers in aquaculture, primarily composed of nitrogen and phosphorus compounds, may also contribute trace elements to the wastewater (Boyd and Massaut, 1999). Consequently, our findings suggest that supplementing additional trace elements to mariculture wastewater during algae cultivation may be unnecessary.

### 3.7 Enhancing diatom cultivation through UV-C irradiation and nutrient regulation

**Figure 7:**
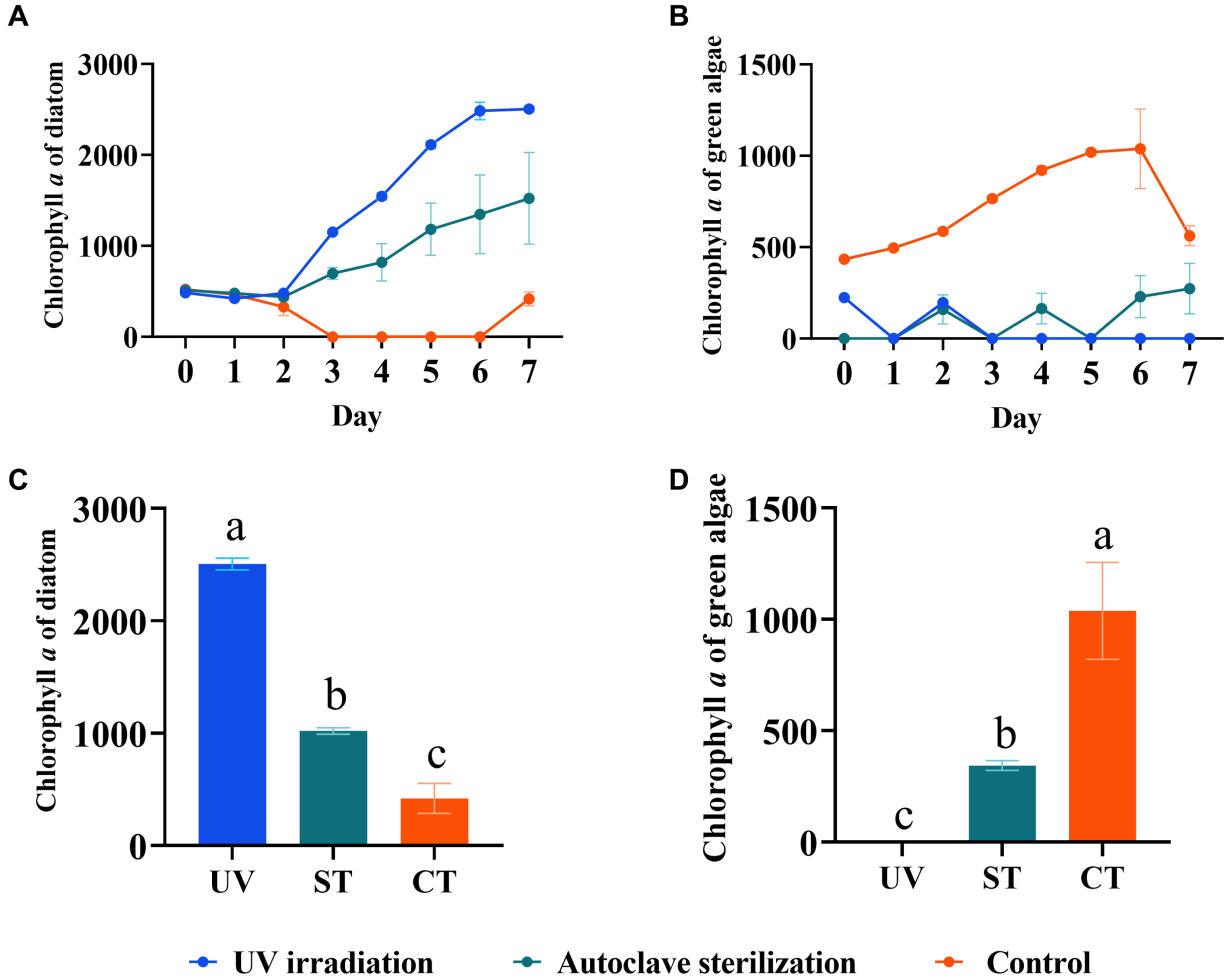
Diatom growth after UV-C irradiation and nutrient regulation. **(A)** Growth trend of diatom after pre-treatments and nutrient regulation. **(B)** Growth trend of green algae after pre-treatments and nutrient regulation. **(C)** Comparison of diatom biomass on day 7. **(D)** Comparison of green algae biomass on day 7. Different letters indicate statistical differences at 0.05 significance level (One-Way ANOVA). UV, UV-irradiation; ST, autoclave sterilization; CT, control.

**Figure 8:**
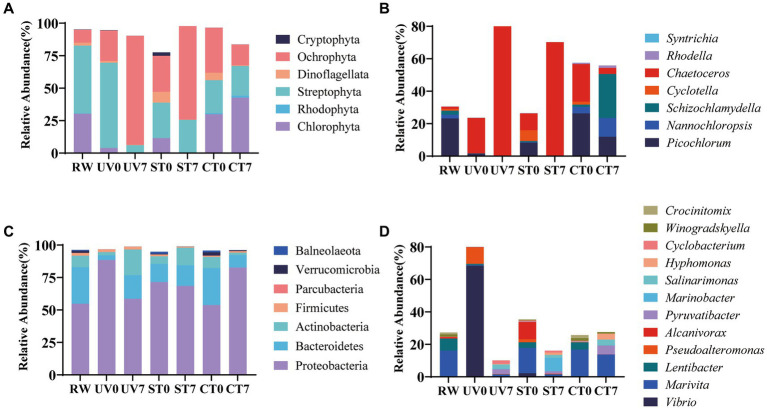
Algal and bacterial community composition at phylum and genus levels. Relative abundance of algae at the phylum **(A)** and species **(B)** levels. Relative abundance of bacteria at phylum **(C)** and species **(D)** levels. RW, raw wastewater; UV, UV-irradiation; ST, autoclave sterilization; CT, control. Only phyla and genera that constitute more than 0.5% percentage of the total in total community were represented.

To develop a practical sterilization technology for diatom targeted cultivation in mariculture wastewater, the effects of UV-C irradiation and autoclave sterilization as pretreatment methods for subsequent diatom growth in wastewater were compared. The results showed that both methods significantly improved diatom growth through the experiment, with the highest diatom abundance observed in the UV-C irradiation group, followed by the autoclave sterilization group, and the least in the control group (*p* < 0.05, Figures 7A,C). In contrast, green algae was suppressed in the UV-C irradiation and autoclave sterilization groups, but flourished in the control group (*p* < 0.05, Figures 7B,D). The 18S rRNA gene analysis revealed that UV-C irradiation and autoclave sterilization groups were dominated by Ochrophyta (particularly *Chaetoceros*), while the control group showed a predominance of Chlorophyta (*Schizochlamydella* and *Picochlorum*) (Figures 8A,B). Both UV-C irradiation and autoclave sterilization significantly decrease the relative abundance of *Marivita* genus (Figure 8D). Interestingly, UV-C irradiation initially increased the relative abundance of *Vibrio* and *Pseudoalteromonas*, which subsequently declined after diatom growth (Figure 8D). These findings indicate that the pre-treatment of mariculture wastewater with UV-C irradiation, in combination with the adjustment of nutrients and addition of exogenous diatoms, significantly enhances diatom growth in mariculture wastewater. This method not only lowers microbial diversity but also minimizes the impact on dissolved trace metals, thereby promoting phytoplankton growth (Passero et al., 2014; Chifflet et al., 2019). Moreover, UV-C irradiation did not completely eliminate native *Chaetoceros* diatom in the wastewater, allowing them to grow alongside the exogenous diatoms after nutrient regulation (Sun et al., 2021). The lower algal and bacterial diversities observed in the treated groups (Figure 8; Supplementary Figure S2) combined with silicate regulation further support the growth advantage for diatoms (Sommer, 1994). However, optimal UV-C irradiation doses and duration for maximizing diatom growth require further investigation. Consequently, UV-C irradiation can replace autoclave sterilization as a pre-treatment method for mariculture wastewater, enabling large-scale wastewater treatment and facilitating diatom targeted culture.

## 4 Conclusion

**Figure 9:**
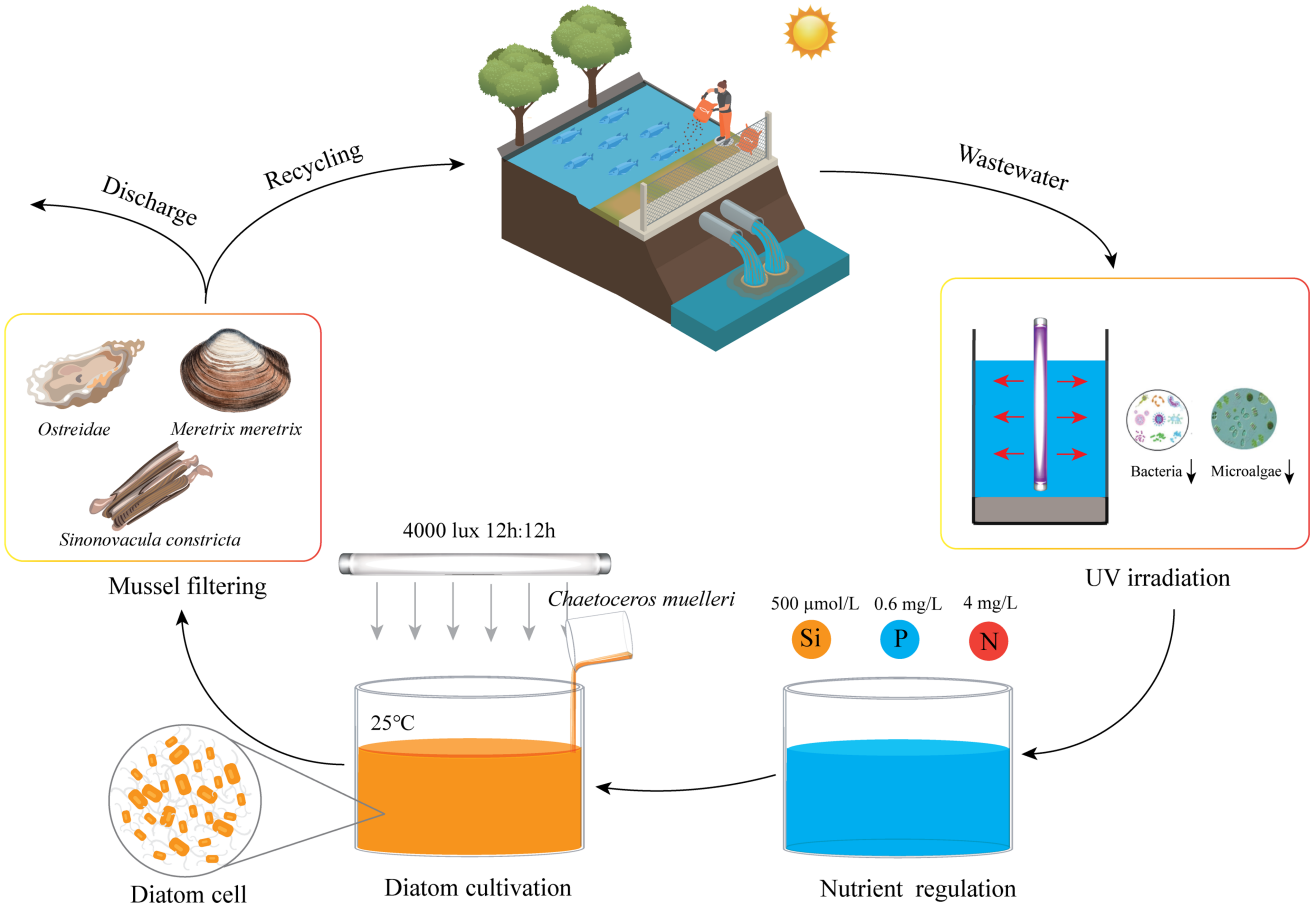
Conceptual model of diatom cultivation in mariculture wastewater. This model illustrates the targeted cultivation of diatoms through UV-C irradiation and nutrient regulation, combined with mollusks for enhanced nutrient recycling.

This study has successfully demonstrated the feasibility of targeted cultivation of the marine diatom *C. muelleri* in mariculture wastewater through precise nutrient regulation. Optimal concentrations of key nutrients, specifically silicate, phosphate, and ammonium, were identified to maximize diatom biomass production. Additionally, this study revealed that trace element supplementation was unnecessary as mariculture wastewater inherently provides adequate amounts. A significant advancement was the use of UV-C irradiation for wastewater pre-treatment, which effectively reduced competition from native microbes during diatom cultivation. This innovative approach not only enhances the efficiency of wastewater treatment but also generates valuable algal biomass suitable for aquaculture feed (Figure 9). Our findings offer practical insights for the widespread adoption of sustainable wastewater bioremediation and resource recovery systems. Further research should focus on pilot-scale implementations and integrating this method with bivalve culture, offering a comprehensive solution to mariculture wastewater management.

## Data availability statement

The datasets presented in this study can be found in online repositories. The names of the repository/repositories and accession number(s) can be found at: https://www.ncbi.nlm.nih.gov/, PRJNA1058243; https://www.ncbi.nlm.nih.gov/, PRJNA1058104.

## Author contributions

JS: Data curation, Formal analysis, Investigation, Writing – original draft. XZ: Conceptualization, Project administration, Supervision, Writing – review & editing. ML: Investigation, Resources, Writing – review & editing. KX: Writing – review & editing. LH: Funding acquisition, Writing – review & editing, Conceptualization, Supervision. ZL: Funding acquisition, Writing – review & editing, Resources.
