# Supplementary material for: Targeted cultivation of diatoms in mariculture wastewater by nutrient regulation and UV-C irradiation
Source: Front Microbiol. 2024 Mar 13;15:1371855. doi: 10.3389/fmicb.2024.1371855 (PMC10976560; doi:10.3389/fmicb.2024.1371855)
Supplement: Supplementary file 1 [file Data_Sheet_1.docx]

**Supplementary Materials for**

**Targeted cultivation of diatoms in mariculture wastewater by nutrient regulation and UV-C irradiation**

Jiacong, Shen^a^, Xiafei Zheng^a, #^, Minhai Liu^a^, Kui Xu^b^, Lin He^a, #^, Zhihua Lin^a^

^a^ Ninghai Institute of Mariculture Breeding and Seed Industry, Zhejiang Wanli University, Ningbo, China

^b^ Hubei Key Laboratory of Edible Wild Plants Conservation and Utilization, Hubei Engineering Research Center of Special Wild Vegetables Breeding and Comprehensive Utilization Technology, College of Life Sciences, Hubei Normal University, Huangshi, China

^#^ Corresponding author: Xiafei Zheng, E-mail: zhengxiafei@hotmail.com; Lin He, E-mail: helin@zwu.edu.cn

**Supplementary Figures and Tables**


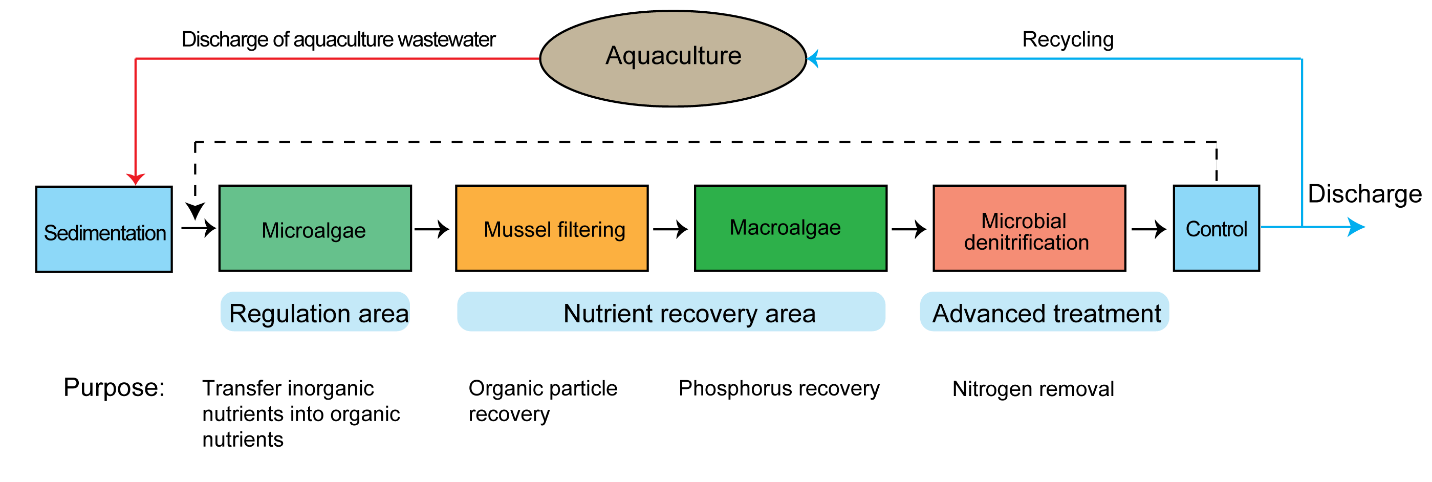


Figure S1 Conceptual diagram of efficient treatment and resource recovery of aquaculture wastewater


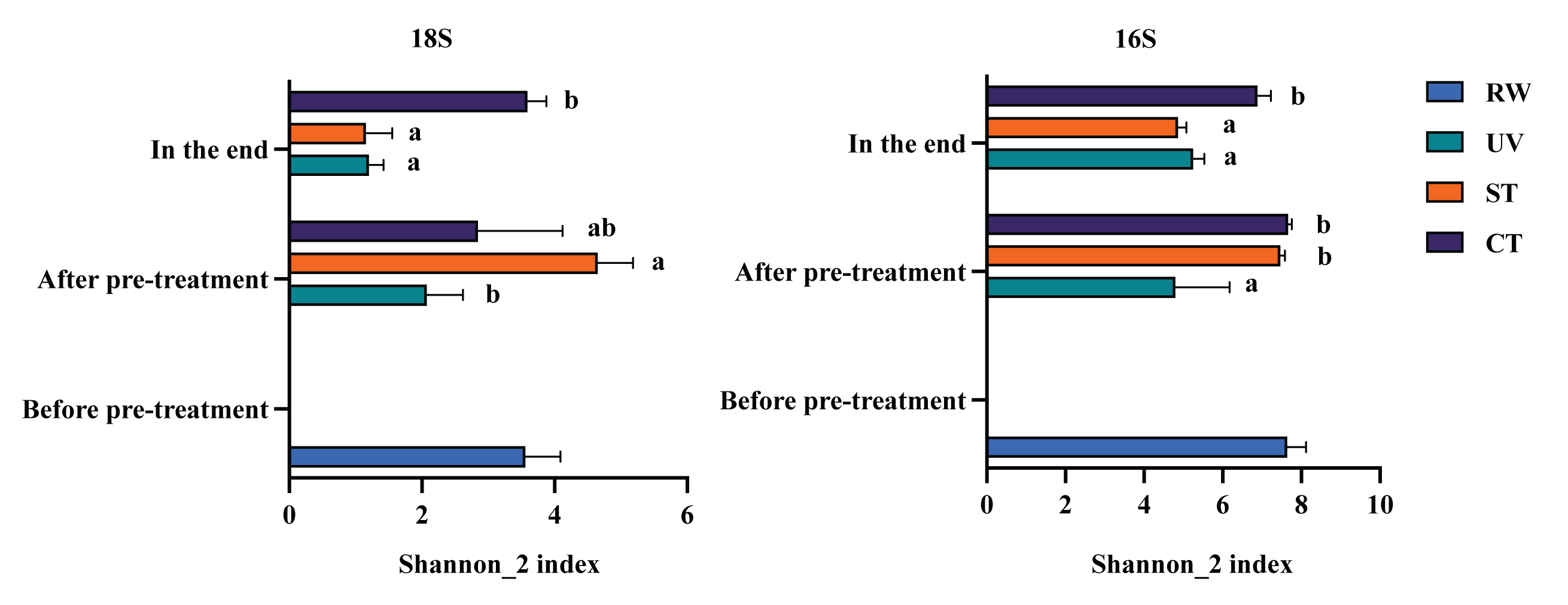


Figure S2 Shannon index of algal and bacterial communities. RW, raw wastewater; UV, UV-irradiation; ST, autoclave sterilization; CT, control.

**Table S1** Nutrient background values for each experimental culture wastewater.

| Parameter | Algae choice | Regulation of silicate | Regulation of phosphate-1 | Regulation of phosphate-2 | Regulation of nitrogen | Different nitrogen source | Regulation of trace elements | UV  irradiation |
| --- | --- | --- | --- | --- | --- | --- | --- | --- |
| Silicate (µmol/L) | 63.8 ± 8.1 | 39.7 ± 4.3 | 28.3 ± 0.5 | 21.7 ± 1.6 | 36.8 ± 0.7 | 26.3 ± 0.6 | 21.7 ± 1.6 | 33.2 ± 0.6 |
| Ammonia (mg/L) | 0.07 ± 0.01 | 3.88 ± 0.08 | 0.31 ± 0.01 | 0.041 ± 0.001 | 0.55 ± 0.02 | 0.14 ± 0.01 | 0.041 ± 0.001 | 0.14 ± 0.01 |
| Nitrite (mg/L) | 0.005 ±0.001 | 1.52 ± 0.03 | 0.307 ± 0.001 | 0.056 ± 0.002 | 0.265 ± 0.008 | 0.053 ± 0.001 | 0.056 ± 0.002 | 0.071 ± 0.003 |
| Nitrate (mg/L) | 0.13 ± 0.01 | 0.88 ± 0.05 | 1.20 ± 0.03 | 0.05 ± 0.01 | 0.54 ± 0.01 | 0.25 ± 0.05 | 0.05 ± 0.01 | / |
| TN (mg/L) | 1.43 ± 0.01 | 7.35 ± 1.15 | 3.98 ± 0.28 | 0.57 ± 0.06 | 1.68 ± 0.20 | 0.27 ± 0.16 | 0.57 ± 0.06 | / |
| Phosphate (mg/L) | 0.083 ± 0.006 | 0.31 ± 0.01 | 0.187 ± 0.006 | 0.36 ± 0.01 | 0.03 ± 0.01 | 0.02 ± 0.01 | 0.36 ± 0.01 | 0.06 ± 0.01 |
| TP (mg/L) | 0.17 ± 0.01 | 0.42 ± 0.04 | 0.23 ± 0.01 | 0.39 ± 0.02 | 0.05 ± 0.01 | 0.03 ± 0.02 | 0.39 ± 0.02 | / |

Data represent as mean ± sd. TN-total nitrogen, TP-total phosphorus.
